# Supplementary figures and images for: Observational assessments of the relationship of dietary and pharmacological treatment on continuous measures of dysglycemia over 24 hours in women with gestational diabetes
Source: Front Endocrinol (Lausanne). 2023 Jan 26;14:1065985. doi: 10.3389/fendo.2023.1065985 (PMC9909093; doi:10.3389/fendo.2023.1065985)

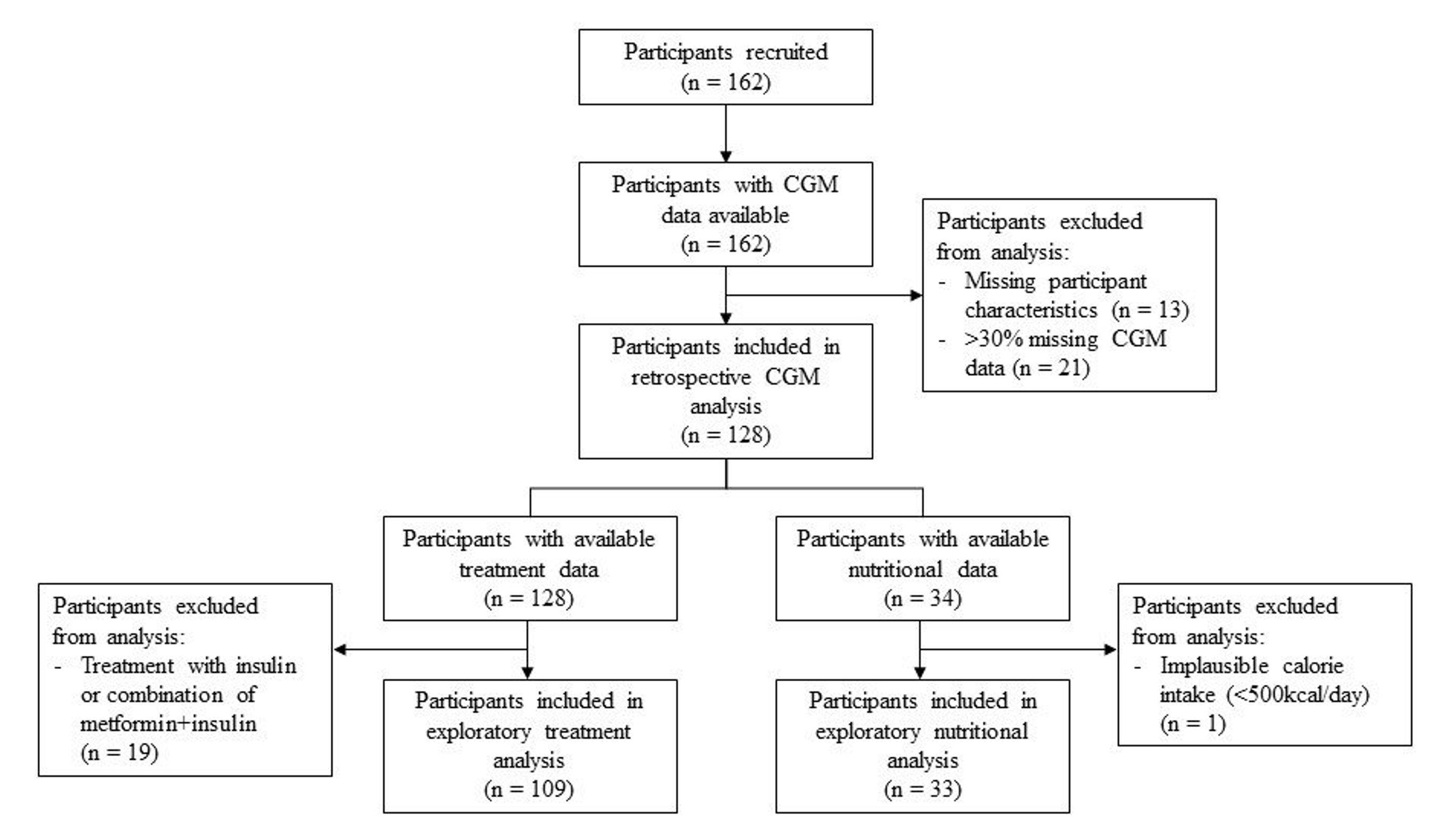

Supplement: Supplementary file 1 [file Image_1.png]
